# A rare association of localized scleroderma type morphea, vitiligo, autoimmune hypothyroidism, pneumonitis, autoimmune thrombocytopenic purpura and central nervous system vasculitis. Case report

**DOI:** 10.1186/1756-0500-5-689

**Published:** 2012-12-20

**Authors:** Fabio Bonilla-Abadía, Evelyn Muñoz-Buitrón, Carlos D Ochoa, Edwin Carrascal, Carlos A Cañas

**Affiliations:** 1 Rheumatology Unit. Fundación Valle del Lili, ICESI University, Avenida Simón Bolívar Cra.98, No.18-49, Cali, Colombia; 2 University of Medical Sciences, La Habana, Cuba; 3 Fundación Valle del Lili, Cali, Colombia; 4 Pathology Unit. Fundación Valle del Lili, and Faculty of Health Sciences, Universidad del Valle, Cali, Colombia

**Keywords:** Localized scleroderma, Morphea, Multiple autoimmune syndrome, Central nervous system vasculitis

## Abstract

**Background:**

The localized scleroderma (LS) known as morphea, presents a variety of clinical manifestations that can include systemic involvement. Current classification schemes divide morphea into categories based solely on cutaneous morphology, without reference to systemic disease or autoimmune phenomena. This classification is likely incomplete. Autoimmune phenomena such as vitiligo and Hashimoto thyroiditis associated with LS have been reported in some cases suggesting an autoimmune basis. To our knowledge this is the first case of a morphea forming part of a multiple autoimmune syndrome (MAS) and presenting simultaneously with autoimmune thrombocytopenic purpura and central nervous system vasculitis.

**Case presentation:**

We report an uncommon case of a white 53 year old female patient with LS as part of a multiple autoimmune syndrome associated with pneumonitis, autoimmune thrombocytopenic purpura and central nervous system vasculitis presenting a favorable response with thrombopoietin receptor agonists, pulses of methylprednisolone and cyclophosphamide.

**Conclusion:**

Is likely that LS have an autoimmune origin and in this case becomes part of MAS, which consist on the presence of three or more well-defined autoimmune diseases in a single patient.

## Background

The localized scleroderma (LS) is distinguished from systemic sclerosis not only by the absence of vasospasm, structural vascular damage, and involvement of internal organs, but also by the distribution of the skin lesions. LS, also known as morphea, presents a variety of clinical manifestations that can include systemic nvolvement [1]. Morphea is characterized by sclerosis of the skin and in some cases underlying tissue. Current classification schemes divide morphea into categories based solely on cutaneous morphology, without reference to systemic disease or autoimmune phenomena. This classification is likely incomplete [2]. Autoimmune phenomena such as vitiligo and Hashimoto thyroiditis associated with LS have been reported in some cases suggesting the possible autoimmune basis of morphea. We report an uncommon case of LS as part of a multiple autoimmune syndrome associated with pneumonitis and central nervous system vasculitis.

## Case presentation

A 53 year old white female patient Jeovah witness was referred to our institution because of general and respiratory symptoms of several days consisting in malaise, weakness, decreased appetite, dyspnea, nonproductive cough, bleeding disorder compatible with idiopathic thrombocytopenic purpura (ITP) and sudden headache associated with diplopia and blurred vision. Her medical history included localized scleroderma type morphea in the lower limbs, vitiligo since 25 years ago, autoimmune hypothyroidism, carpal tunnel and obesity.

**Figure 1:**
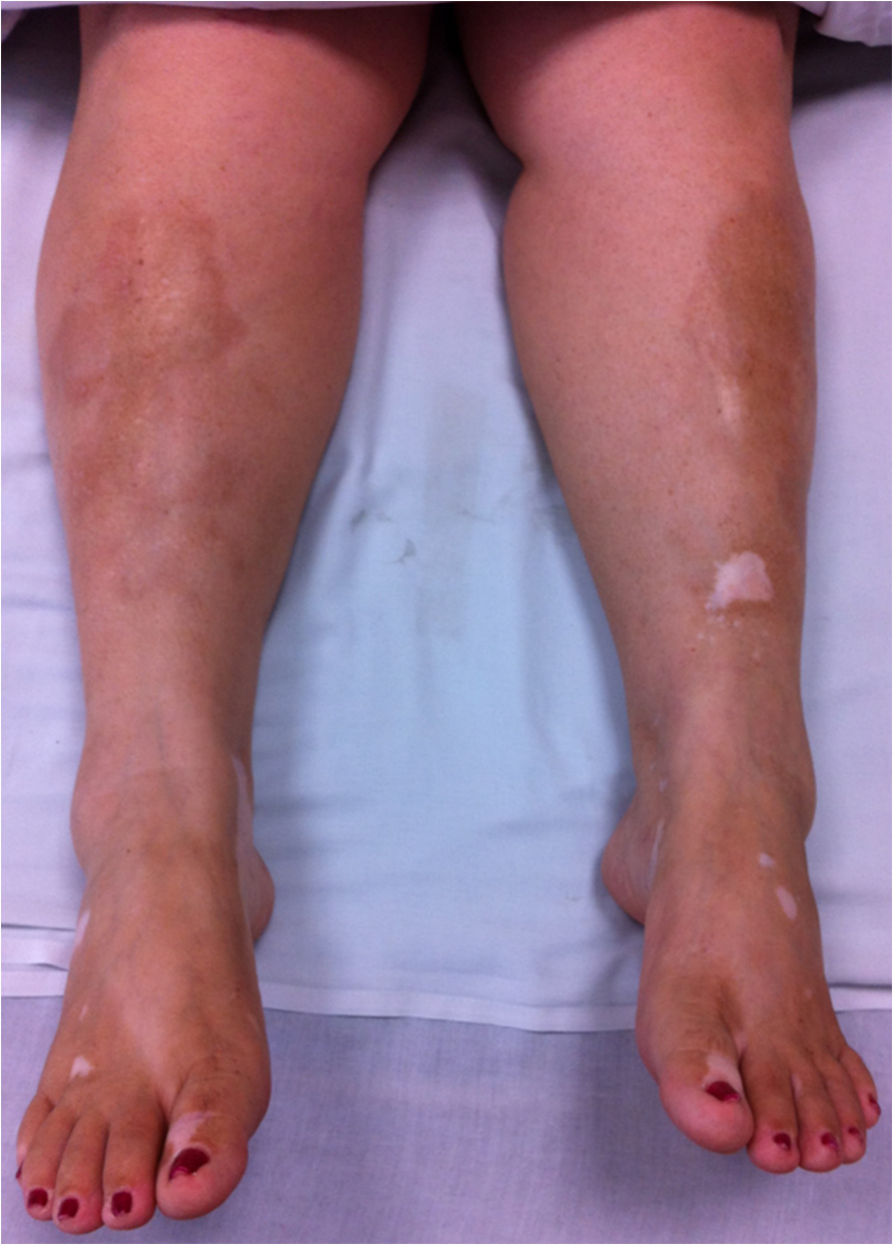
Vitiligo and morphea lesions on the lower limbs.

**Figure 2:**
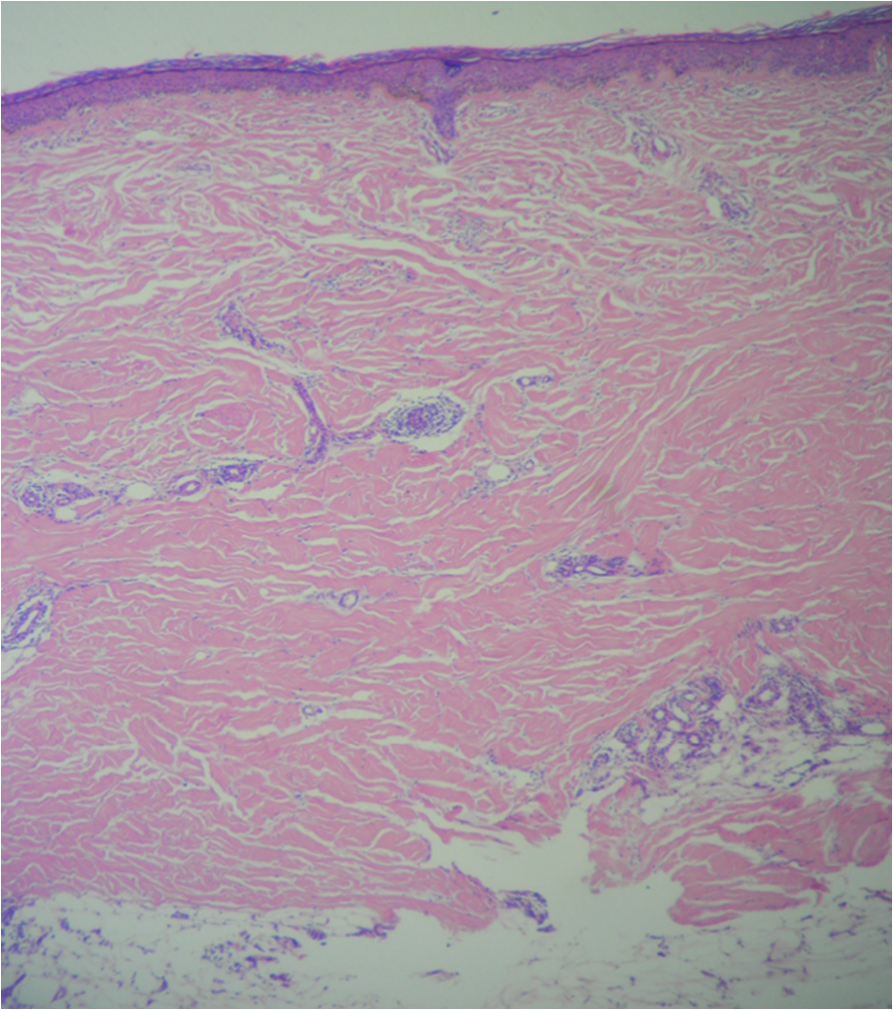
Histopathologic examination of the morphea lesions, showing interstitial inflammation and the homogenization of collagen (hematoxylin and eosin stain, magnification x100).

**Figure 3:**
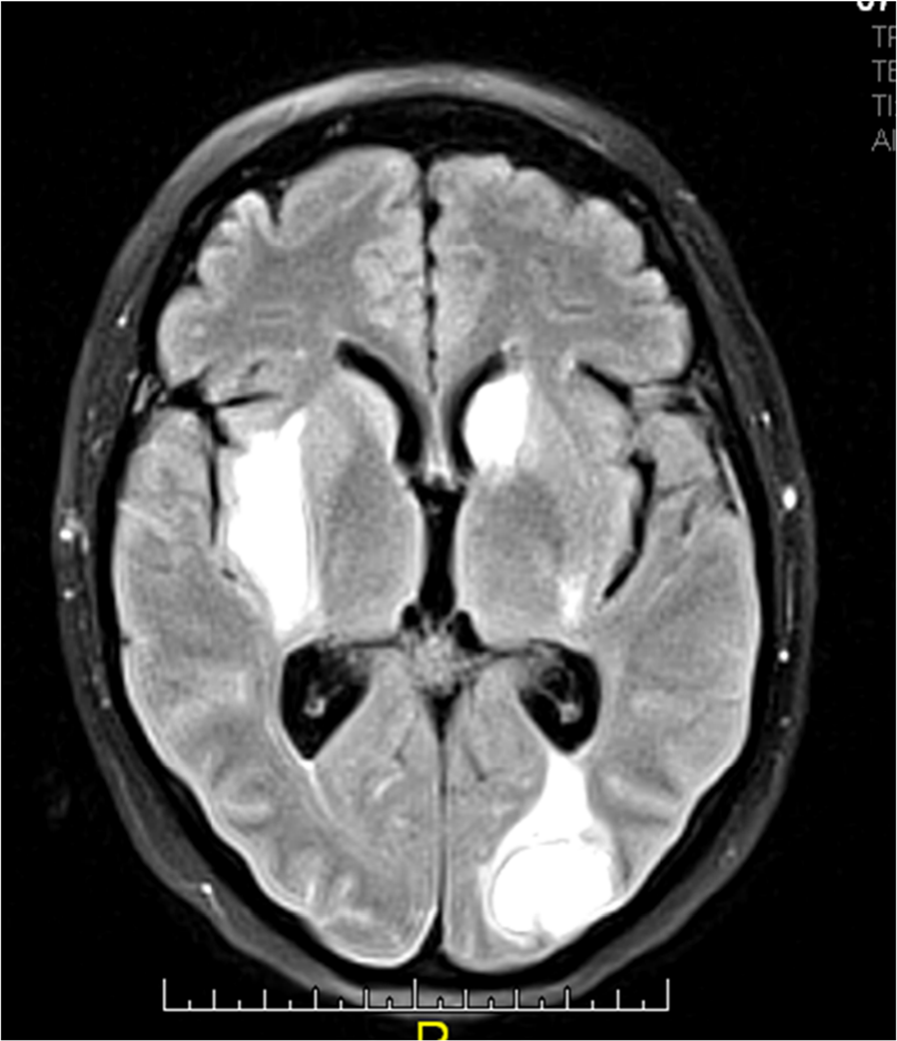
MRI brain showed multiple cerebral infarctions with hemorrhagic transformation.

**Figure 4:**
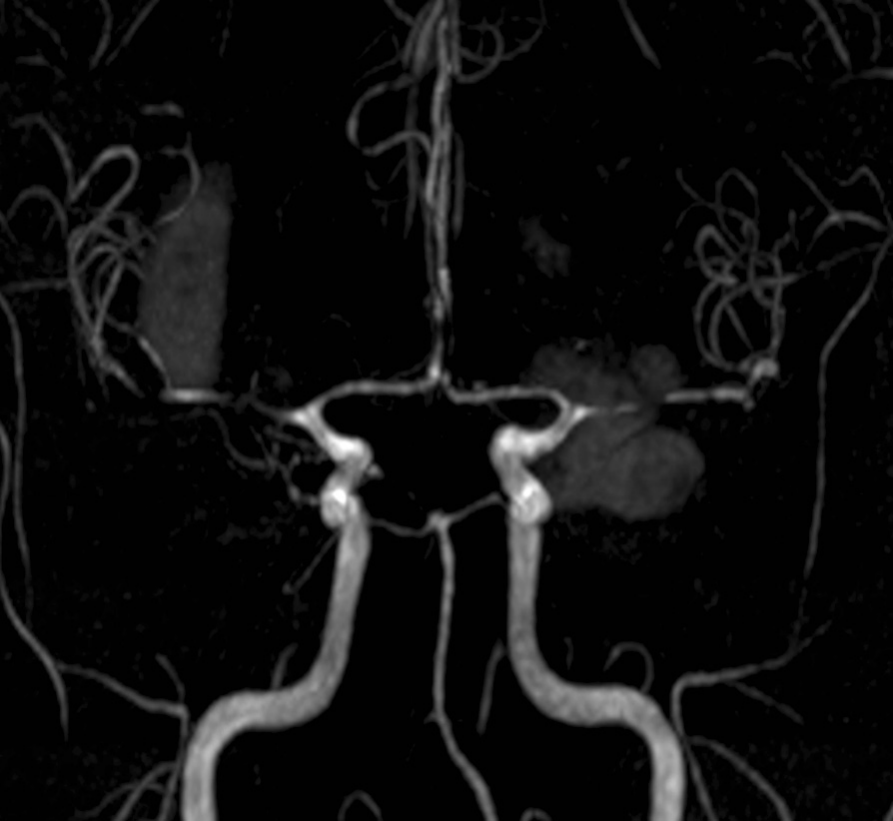
Brain magnetic resonance angiography showed irregularity in the pattern of cerebral arteries in "beads".

At physical examination on admission, her cardiopulmonary and abdominal examination was normal, no evidence of oral ulcers, alopecia, malar rash, synovitis, sclerodactyly, Raynaud phenomenon or dry symptoms. Hypopigmented lesions of vitiligo were observed in the lower limbs associated to atrophic violaceous plaques (Figure 1), these last lesions showing interstitial inflammation and homogenization of collagen in the histopathology study which was consistent with morphea (Figure 2). The neurological examination revealed diplopia and mild dysmetria. Initial studies showed pulmonary infiltrates and lung nodules of unclear etiology by X-ray and chest tomography. A fiberoptic bronchoscopy was performed evidenced only erythema of mucosa with negative bacteriological cultures. Echocardiogram was reported as normal. Laboratory test reported negative BK serial sputum. Clotting studies, renal function, C-reactive protein, bilirubin level and urine analysis were normal. Leukocytes count of 9830 cells/mm3 with normal hemoglobin and differential count and thrombocytopenia in the range of 41.000 which limited performing lung biopsy. Aspirate and flow cytometry in bone marrow were reported as normal, and there was no evidence of schistocytes in peripheral blood spread. Abdominal ultrasonography not demonstrated splenomegaly. Secondary infectious (HIV, hepatitis B and C, blood and urine cultures) and drug-related causes of thrombocytopenia were ruled. A brain computed tomography (CT) reported right and left gangliobasal and left lamellar intraventricular hemorrhage with ventricular drainage without evidence of arteriovenous malformation. Pulses of methylprednisolone were initiated in the context of intracerebral bleeding and autoimmune thrombocytopenia with unfavorable response and due to the inability of transfusion, hyperimmune human gamma globulin (0,5 gr/kg/day) was indicated. Refractoriness was presented to the application of gamma globulin and so it was necessary to use thrombopoietin receptor agonists (Eltrombopag) with a slow good response. Complementary autoimmune laboratory showed C3 136 mg/dl (90–180 mg/dl), C4 18 mg/dl (10–40 mg/dl), direct Coombs test, rheumatoid factor, Scl70, lupic anticoagulant, ANAs, AntiDNA, antineutrophil cytoplasmic antibodies (ANCAS) and anticardiolipins titles within normal limits, as well as extractable nuclear antigens (anti-Ro 7.7 U (<20), anti-La 2.7 U (<20), anti-Sm 1.9 U (<20), and anti-RNP 4.8 U (<20)). The antimicrosomal (45 UI/ml) and anti-thyroglobulin antibodies (21 UI/ml) were positive with TSH in normal range. Control lung imaging showed improvement of pulmonary involvement. Persistent headache, photophobia, nausea, emesis and diplopia made it necessary the realization of a cerebral magnetic resonance angiography that reported multiple cerebral parenchymals infarcts (right external capsule, left head caudate nucleus and bilateral occipital) with hemorrhagic transformation (Figure 3). Irregularity in cerebral arteries in "beads" pattern compatible with central nervous system (CNS) vasculitis was observed (Figure 4). Intracranial neoplasms of a primary and secondary nature, aneurysms and vascular malformations were ruled out by this study. A diagnosis of cerebral vasculitis was made in the light of findings requiring initiation of intravenous steroids (methylprednisolone 1 gr/day per five days) and intravenous cyclophosphamide (1 gr monthly) with clinical improvement and satisfactory recovery.

## Discussion

LS associated with vitiligo and another autoinmune phenommena like Hashimoto’s thyroiditis and autoimmune thrombocytopenic purpura have been rarely reported to occur simultaneously with evidence of improvement to treatment with systemic steroids [3-6]. Some studies have also indicated an association of autoimmune diseases and an increased frequency of serum autoantibodies with morphea [7]. In the literature, it has been raised that this association is more than coincidence, and it suggests an autoimmune basis for these conditions [8]. In other studies, elevated serum cytokines and cell adhesion molecules was related to the immune activation of localized scleroderma [9], as well as decreased regulatory T cells in patients with morphea contributing to loss of tolerance seen in this and other autoimmune diseases [10,11]. Also some non–organ-specific autoimmune conditions like systemic lupus erythematosus [12,13], rheumatoid arthritis [14] and necrotizing vasculitis [15] have been reported associated with LS, in the latter case requiring the use of systemic steroids and cyclophosphamide with gradual improvement. Central nervous system (CNS) involvement and ophthalmologic manifestations in LS has been reported by several authors and, particularly, in linear scleroderma (en coup de sabre) and in progressive facial hemiatrophy [2,16] and a report of LS associated with progressing ischemic stroke where it is thought that was caused by hemodynamic disturbances from localized sclerotic obstruction of the middle cerebral artery [17], however there are not clearly reports of association between morphea and central nervous system vasculitis. Table 1 summarizes some of the reports of LS associated with autoimmune diseases and the presence of autoantibodies. Is likely that LS have an autoimmune origin and in this case becomes part of a multiple autoimmune syndrome (MAS), which consist on the presence of three or more well-defined autoimmune diseases in a single patient [18]. To our knowledge this is the first case of a morphea forming part of a multiple autoimmune syndrome and presenting simultaneously with ITP and CNS vasculitis.

**Table 1:** LS associated with autoimmune diseases, presence of autoantibodies, treatment and response

**Case**	**Age, sex**	**Morphea’s location**	**Associated autoimmune condition**	**Positive autoantibodies**	**Treatment**	**Response to treatment**	**Ref.**
1	48-year-old woman	Plaques over the trunk	Vitiligo, Hashimoto’s thyroiditis	Antithyroglobu- lin antibody, anti-thyroid peroxidaz	Not described	Not described	3
2	16-year-old girl	On her trunk and right arm.	Necrotizing vas- culitis of arterioles within the subcutaneous fat and muscles. Mononeuritis multiplex.	Lupus anticoaglant test, Anti-DNA antibody, (LE) test, Anticardiolipin-β2GPI antibody	Pulse therapy with 1 g methyl prednisolone for 3 days, cyclophosphamide (50 mg/d).	Gradually improved	13
3	16-year-old girl	Umbilical region, right hemi thorax and back of the right hand	Idiopathic thrombocytopenic purpura	ANAS, Anti DNA	Not described	Not described	6
4	22-year-old woman	Anterior chest	Idiopathic thrombocytopenic purpura	None	Not described	Not described	6
Our case	53 year old woman	Atrophic violaceous plaques in the lower limbs	Vitiligo, autoimmune hypothyroidism, neumonitis, autoimmune thrombocytopenic purpura and central nervous system vasculitis	Anti-microsomal and Anti-thyroglobulin antibodies	Methylprednisolone 1 gr/day per five days) and intravenous cyclophosphamide (1 gr monthly	Clinical improvement	

## Conclusion

Is likely that LS have an autoimmune origin supported by the reports described and its association with other autoimmune diseases, in this case forming part of MAS and with the aggravation to be associated with life-threatening conditions requiring aggressive treatment. This makes it important to look for systemic conditions in patients with localized scleroderma that may worsen the clinical localized disease.

## Consent

Written informed consent was obtained from the patient for publication of this manuscript and accompanying images. A copy of the written consent is available for review by the Editor-in-Chief of this journal.

## Competing interests

The authors declare that they have no competing interests.

## Authors' contribution

CO and EM analyzed and interpreted the patient data regarding the rheumatology disease and the treatment. EC performed the histological examination of the skin. FB and CC were the major contributors in writing the manuscript. All authors read and approved the final manuscript.
